# The Influence of Educational Psychology on Modern Art Design Entrepreneurship Education in Colleges

**DOI:** 10.3389/fpsyg.2022.843484

**Published:** 2022-06-27

**Authors:** Huan Zhao, Shuyi Li, Hui Xu, Lele Ye, Min Chen

**Affiliations:** ^1^College of Zhang Daqian's Fine Arts, Neijiang Normal University, Neijiang, China; ^2^School of Arts & Communication, Beijing Normal University, Beijing, China; ^3^Homerton College, University of Cambridge, Cambridge, United Kingdom; ^4^Zhijiang College of Zhejiang University of Technology, Shaoxing, China; ^5^School of Business, Wenzhou University, Wenzhou, China

**Keywords:** educational psychology, art psychology, college, modern art design, entrepreneurship education

## Abstract

The purpose of this study is to analyze the relationship between psychology and modern art design education in colleges, and to provide the basis for improving the quality of art education in colleges. Based on the relevant theory, the characteristics of educational psychology and art psychology and the correlation between them were analyzed from the angle of art education. According to the characteristics of college students' psychological development, the significance of art psychology-related courses was analyzed. Through the form of questionnaire, a total of 200 students (400 students in total) were randomly selected from Xi'an Academy of Fine Arts (professional art college) and Xi'an University of Science and Technology (comprehensive university), respectively. A total of 400 questionnaires were issued, and 382 valid questionnaires were recovered. The results show that almost all students in two colleges think pedagogy and educational psychology are the theoretical basis for art teachers, while less than one-third of students in Xi'an University of Science and Technology think that art psychology is the theoretical basis for art teachers. The difference between the students' aesthetic and life values in the two colleges is significant (*p* < 0.01). In the 15 directions of life values, there are significant differences in five directions: spirit—material (*p* < 0.05), enrichment—emptiness (*p* < 0.05), enthusiasm—apathy (*p* < 0.05), hope—despair (*p* < 0.01), and dedication—acceptance (*p* < 0.01). It shows that the psychological and values of college students are changeable, and the educators should pay attention to the education in the related fields of psychology. This exploration was conducted based on educational psychology, which is of great significance for improving the educational level of art psychology in colleges and enable students to form correct aesthetic standards and life values.

## Introduction

In the process of the overall development of Chinese education, art design education has gone through twists and turns. At the end of the 20th century, China decided to promote quality education in policy, and as the goal of overall development education, art design education was more stable. In the past 6 years, its development entered a prosperous era (Mcgowan and Bell, 2020; Lou and Li, 2021). Especially at the end of 2013, the Third Plenary Session of the 18th Central Committee of the CPC proposed that “deepening the comprehensive education reform” is to improve the art education in the teaching process and improve the aesthetic and humanistic quality of students. It played an important role in infiltrating the missing art education into classroom teaching and improving the students' aesthetic and humanistic quality (Hu and Zhang, 2016; Verboom et al., 2017). At the end of 2015, the general office of the State Council issued the *Report on Strengthening Scientific and Technological Innovation in An All-round Way* and put forward some suggestions on how to build a scientific art education curriculum system. In the same year, art design education was incorporated into the *Education Law* and the *Higher Education Law*. Under the protection of law, the arts design education of colleges became an education that must be implemented. In 2019, the Ministry of Education issued *Opinions on Strengthening the Art Education in Colleges in the New Era*, forming a new arrangement and planning of art design education in colleges, and clearly pointed out that the education forms must be diversified, and the high-quality and socialist modern university art design education system with Chinese characteristics should be formed by 2035.

Although some policies pay attention to art education in colleges, there are still many problems in art education in colleges, such as incomplete curriculum, lack of top-level design, few types of art design education available, students' vague concept of aesthetic education, not familiar with the content of art design education and not interested in traditional art (Barnes and Smagorinsky, 2016). More importantly, colleges have little understanding and guidance of students' psychological changes in the process of art learning (Wu et al., 2020). Throughout the psychological contents involved in worldwide art and design education, the famous psychologist Rudolf Arnheim served as the president of the American Aesthetic Society twice and made great achievements in the field of art psychology, with many work and extensive problems. The Gestalt psychology theory put forward is more suitable for the interpretation of art forms. Heterogeneous isomorphism theory is also used to explain the possibility that visual artworks become the perceptual experience of observers and the unique perceptual mode of artists (D'Angeli et al., 2016; Katsur, 2018). In China, although Ding Ning's art psychology has filled the gap in the field of Chinese art psychology (Yao et al., 2016) and The Guangzhou Academy of Fine Arts opened an experimental course of art psychology, it still fails to make up for the lack of formal psychology education for art majors (Jia et al., 2017). However, the development of art psychology still has a broader prospect, and countless scholars are looking forward to the further success of art design education. The prosperity and development of the cultural industry are inseparable from the cultivation of innovative and entrepreneurial art talents. Conforming to the wave of innovation and entrepreneurship education and cultivating the innovation and entrepreneurship consciousness, innovation and entrepreneurship spirit, and innovation and entrepreneurship ability of art talents are the major historical mission faced by contemporary Chinese academy of fine arts. The purpose of innovation and entrepreneurship education in the academy of fine arts must return to people themselves. It is not only to obtain utilitarian economic benefits, but also to highlight the subject status of students, reflect their dignity and value, and cultivate talents with balanced and coordinated development of high rationality and high emotion for the society.

Based on this, according to the correlation between educational psychology and art psychology and theoretical basis, the psychology-related courses, students' aesthetic and life values of professional art colleges and comprehensive university were analyzed creatively through the form of questionnaire, which provides practical basis for improving modern art design education in colleges.

## Theoretical Basis and Research Design

### Educational Psychology and Art Psychology From the Perspective of Art Education

Educational psychology is a kind of social psychology that studies people's learning, the effect of educational intervention, teaching psychology, and school organization in educational context. The research focus of educational psychology is to apply psychological theories or research results to education. Educational psychology can be used to design courses, improve teaching methods, improve learning motivation, and help students to face the difficulties and challenges in the process of growth (Bernardo et al., 2018; Wu et al., 2019). Educational psychology is an interdisciplinary subject with distinct characteristics. Table 2 is the main manifestation of its interdisciplinary characteristics.

**Table 1:** Characteristics of educational psychology.

**Characteristic type**	**Content**
Interdisciplinary nature	The intersection of psychological science and educational science; the intersection of basic science and applied science; the intersection of natural science and humanities.
Wide application	Educational psychology can be used to design courses, improve teaching methods, stimulate learning motivation, and help students face the difficulties and challenges in the process of growth.

**Table 2:** The significance of offering art design education courses in colleges.

**Different directions**	**Concrete content**
Developing sensory system	College students can actively mobilize the five senses of vision, hearing, touch, taste and even smell (Chun et al., 2016), intuitively feel all kinds of beautiful things; they can perceive the media of art design works as a whole, and grasp the formal analysis of the association and connection between various elements and components in art design works.
Developing the neural system of aesthetic cognition	They have rich aesthetic schema, can consciously remember the beautiful things they feel, can actively construct the memory of aesthetic information, can carry out aesthetic imagination and association activities, search the aesthetic information stored in the brain, process the images, and connect, transform or reorganize the images (Grady and Porche, 2019).
Developing the emotional system of aesthetic experience	They can actively appreciate all kinds of beauty, such as classical beauty, popular beauty and traditional beauty (Benovsky, 2016); they have strong aesthetic experience ability and strong aesthetic consciousness, and can appreciate beauty of different styles; they can distinguish beauty from ugliness, and have positive aesthetic value and life value; they can appreciate Chinese traditional culture, and distinguish the charm of traditional cultural value.
Developing the movement system of aesthetic performance skills	They can mobilize all kinds of emotions (Rogoza et al., 2018) and thoughts for aesthetic performance, and have more systematic skills to beautify the appearance and mind. They have 2–3 types of high-level aesthetic performance skills, and can combine body aesthetic performance with skills.
Aesthetic creation and the development of whole brain and whole body	They have rich aesthetic images, can use certain skills, follow the essence and laws of beauty (Vasiliki et al., 2017), and create things with higher aesthetic images; they can learn cross-border thinking, get rid of professional limitations, and understand the common points of beauty (Wu et al., 2019).

Art psychology is an applied psychology for art research after experimental aesthetics. It is a special psychology which aims at and applies to the field of fine arts, and it is also the application of psychology in the field of fine arts (Agocs, 2018). In fact, art psychology is a kind of psychology to explore the composition of art with people's creative emotion. Art psychology is to study art works and human psychology to lay a foundation for better study of art. Therefore, it is closely related to art itself and art education. More directly, art psychology can help guide artists' works to be better understood (Ruta et al., 2021). Therefore, based on educational psychology, the influence of art psychology on modern art education in colleges is analyzed.

### The Relationship Between Educational Psychology and Art Psychology

Art psychology and educational psychology have something in common. They belong to the category of applied psychology; they all belong to psychology; they all have the same point of view to study the laws of psychology; they all have substantive significance and are used as indexes and methodology in practical activities. The former is unique and the latter is universal, so its functions are different. In *Educational Psychology*, there is a famous saying, “The foundation of any teacher's profession is educational psychology.” Art psychology plays a more important role in art design education. It can be understood as: art psychology is the foundation of any art design educator, art-related workers and artist (Kime, 2016).

For the relationship between art education and art psychology, art design education is based on art discipline. Its purpose is to develop art knowledge and skills, meet the needs of human society, economy, spirit and culture, improve people's personality, form people's basic art quality, and promote people's all-round development. The idea of art design education is to live in the present, and let art penetrate into life, into thinking, into the soul (Tanja et al., 2016). Art education decides art psychology, which is born for art design education and serves for art design education.

### Innovation and Entrepreneurship Education System for Students in Academy of Fine Arts

The academy of fine arts implements education through emotional experience or subtle influence. The creation and appreciation of art works will be accompanied by individual strong emotions. Students' creative passion can be triggered through the infiltration of emotional force. Artistic imagination is the promotion of college students' innovation and entrepreneurship ability. Art education can improve college students' non-logical thinking abilities such as intuitive thinking and image thinking, and promote the cultivation of college students' innovation and entrepreneurship ability. Creative thinking is the unity of image thinking and abstract thinking. Image thinking is very crucial for the cultivation of innovation and entrepreneurship ability, which is the essential feature of artistic thinking.

From the perspective of the employment orientation of art students, college students of the academy of fine arts have active thinking and advocate freedom. When planning their future careers, unlike other ordinary college students who focus on stability, they tend to choose the majors they are interested in or occupations with strong freedom. Hence, many art college students have tried various entrepreneurial attempts related to their majors during their school years. Most entrepreneurs choose creative industries such as advertising design, media, film and television, art photography, and creative product development.

The innovation and entrepreneurship education system of academy of fine arts includes four dimensions: external environment system, internal operation system, education and teaching system, and operation guarantee system. The internal operation system of innovation and entrepreneurship education is the key to the content of the whole system. The hierarchical curriculum system of innovation and entrepreneurship education in academy of fine arts has been constructed. The course can be divided into two modules: theoretical knowledge and practical skills. Four sub-modules can be set under these two modules: innovation and entrepreneurship knowledge, professional knowledge, professional practice, and innovation and entrepreneurship ability improvement. The goal of the first level of innovation and entrepreneurship education is to cultivate art talents with good innovation and entrepreneurship quality. A broad-spectrum innovation and entrepreneurship education curriculum system that takes into account general education and professional education must be established. According to the second level goal of art talent training, the creators of their own jobs are trained. The focus of this stage is to cultivate students' entrepreneurship and some relevant courses such as innovation and entrepreneurship knowledge and innovation and entrepreneurship practice can be set up. Based on the existing curriculum modules, according to the needs of social and economic development and the personalized needs of students in academy of fine arts, innovation and entrepreneurship intensive courses can be set for students to choose, so as to form a key innovation and entrepreneurship education curriculum system of “general education + professional education + intensive education.”

### The Significance of Art Psychology Related Courses

#### The Characteristics of Psychological Development of College Students

The characteristics of psychological development of college students are mainly manifested in three aspects, namely, cognitive development, emotion development, and aesthetic psychology (Masland and Lease, 2016; Peltz and Raymond, 2016). The characteristics of cognitive development of college students are mainly reflected in the development level of intelligence factors such as perception, attention, memory, observation ability, and thinking ability. The general trend of college students' emotion is to transform from external performance to internal experience. Because emotion is controlled by autonomic nerve in physiological mechanism, the direct reason for emotion is people's judgment and evaluation of the things that act on it. Obviously, emotion and cognitive activities are inseparable. The mark of emotional development level is mainly reflected in the relationship between emotion and rationality (Qian et al., 2018; Chen, 2019).

In the aspect of aesthetic psychology, college students combine aesthetic feeling with sociology, psychology, anthropology, and other disciplines, which is an open tendency. It tries to get rid of the passive position and is more willing to carry out aesthetic re-creation activities in the process of aesthetic appreciation. The dialectical aesthetic tendency of art design works is analyzed from a dialectical perspective (Flynn, 2016; Lin, 2016).

#### Significance of Art Design Education Curriculum in Colleges

**Figure 1:**
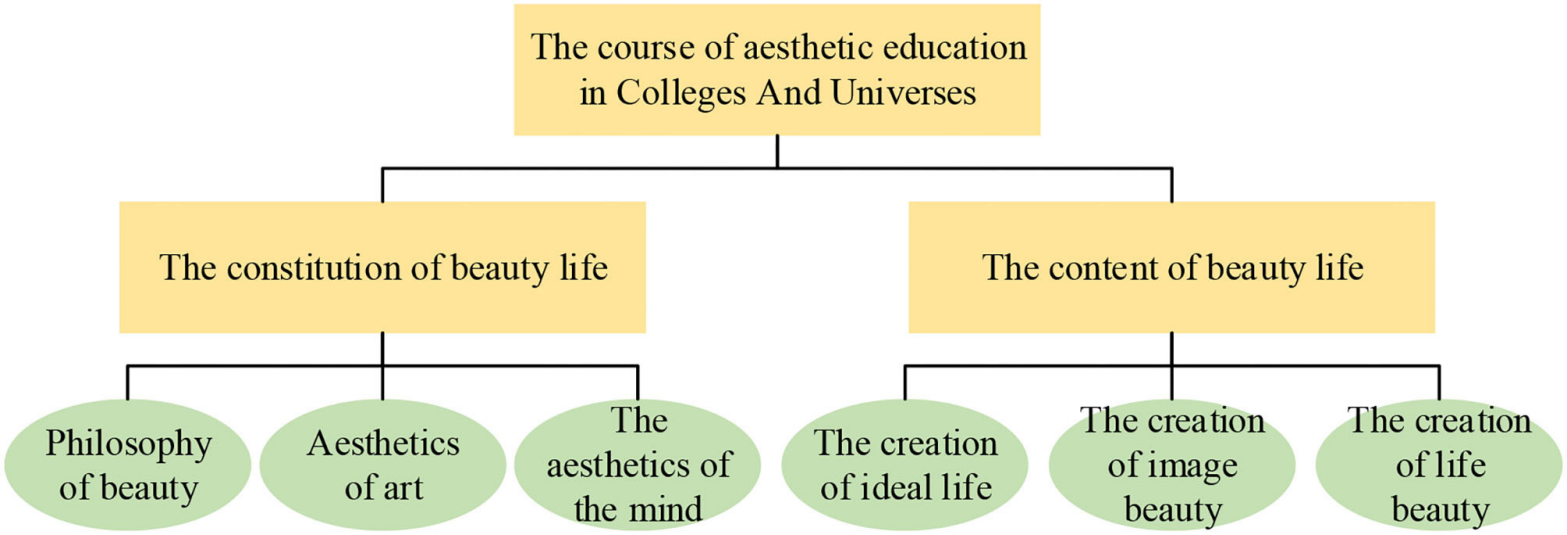
Contents of art design education course in colleges.

Figure 1 shows the art design education course in colleges.

Table 2 shows the significance of offering art design education courses in colleges.

### Research Project Design

(1) In the form of questionnaire, 200 students were randomly selected from Xi'an Academy of Fine Arts (professional academy of fine arts) as well as Xi'an University of Science and Technology (Comprehensive University). A total of 400 questionnaires were distributed and 382 valid questionnaires were recovered, with an effective recovery rate of 95.5%. The questionnaire had two scales. Three questions were set in the questionnaire.

Q_1_: What is the theoretical basis of education that a qualified or even excellent art design teacher should have?

A: educational psychology, B: pedagogy, C: art design education, D: arts psychology.

Q_2_: Have you studied educational psychology?

A: understand, B: have studied, C: have heard, D: have never heard.

Q_3_: Have you studied art psychology?

A: understand, B: have studied, C: have heard, D: have never heard.

With the relevant value scales in China and foreign countries as the reference, the questionnaire of college students' life value was self-compiled, which included three dimensions—“life purpose,” “life attitude” and “life style.” Each dimension contained five directions, and each pair of directions was composed of two extreme words. They were divided into five equal distances, from positive to negative. The total distance was 3 points, and each equal distance was 0.6 points. According to their own actual situation, the research objects chose one of the five equal distances, ticked “√,” then converted the statistical times of each group into scores, and added them to get the scores of each group in each direction.

As a tool of aesthetic ability test, the questionnaire was composed of 8 questions. The answers to each question were scored by two professional teachers, and the average score was taken as the actual score of the test.

(2) Confirmatory factor analysis was used to test the construct validity of the questionnaire. Cronbach a value was used to test the reliability of the measurement (Frielink et al., 2016). Independent sample *t*-test analysis and one-way ANOVA (Ruthig et al., 2017) were used to test the influence of college art design education curriculum on students' life value and aesthetic ability.

(3) The SPSS26.0 statistical software was used to analyze the collected questionnaire data, and Origin 2018Bit was used for data visualization.

## Results and Analysis

### Analysis Results of the Reliability and Validity of the Questionnaire

**Figure 2:**
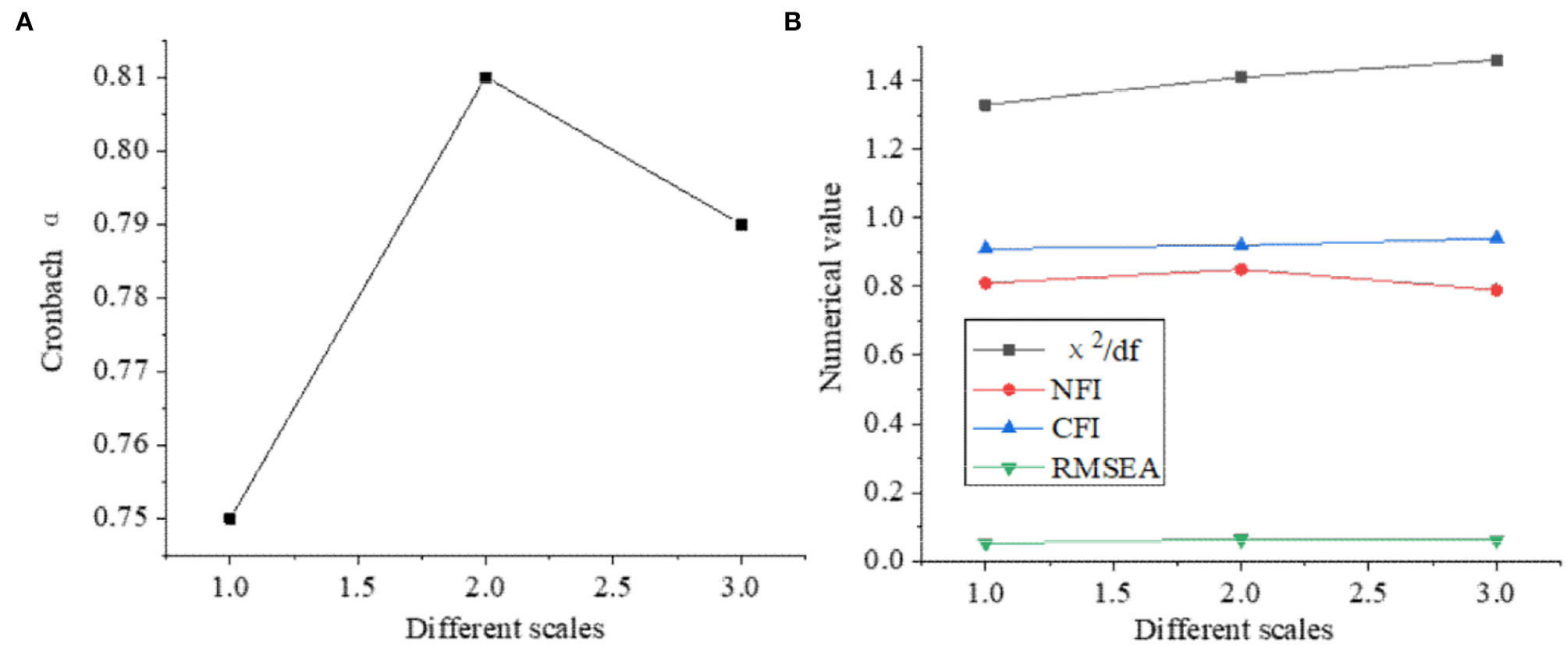
The results of reliability and validity analysis of questionnaire **(A)** reliability, **(B)** validity.

The reliability and validity of the designed questionnaire were analyzed, as shown in Figures 2A,B.

Figure 2 shows that Cronbach α values of each scale are between [0.75, 0.81], indicating that the reliability of the scales has reached the ideal value. The χ^2^/df values of each scale are <2, and the RMSEA of the three questionnaires is between [0.05, 0.1], which indicates that the scale has a good fitting ability to the data and can be used in the investigation and analysis of this study.

### A Comparative Study of the Basic Courses of Art Design Teachers' Education Theory in Colleges

**Figure 3:**
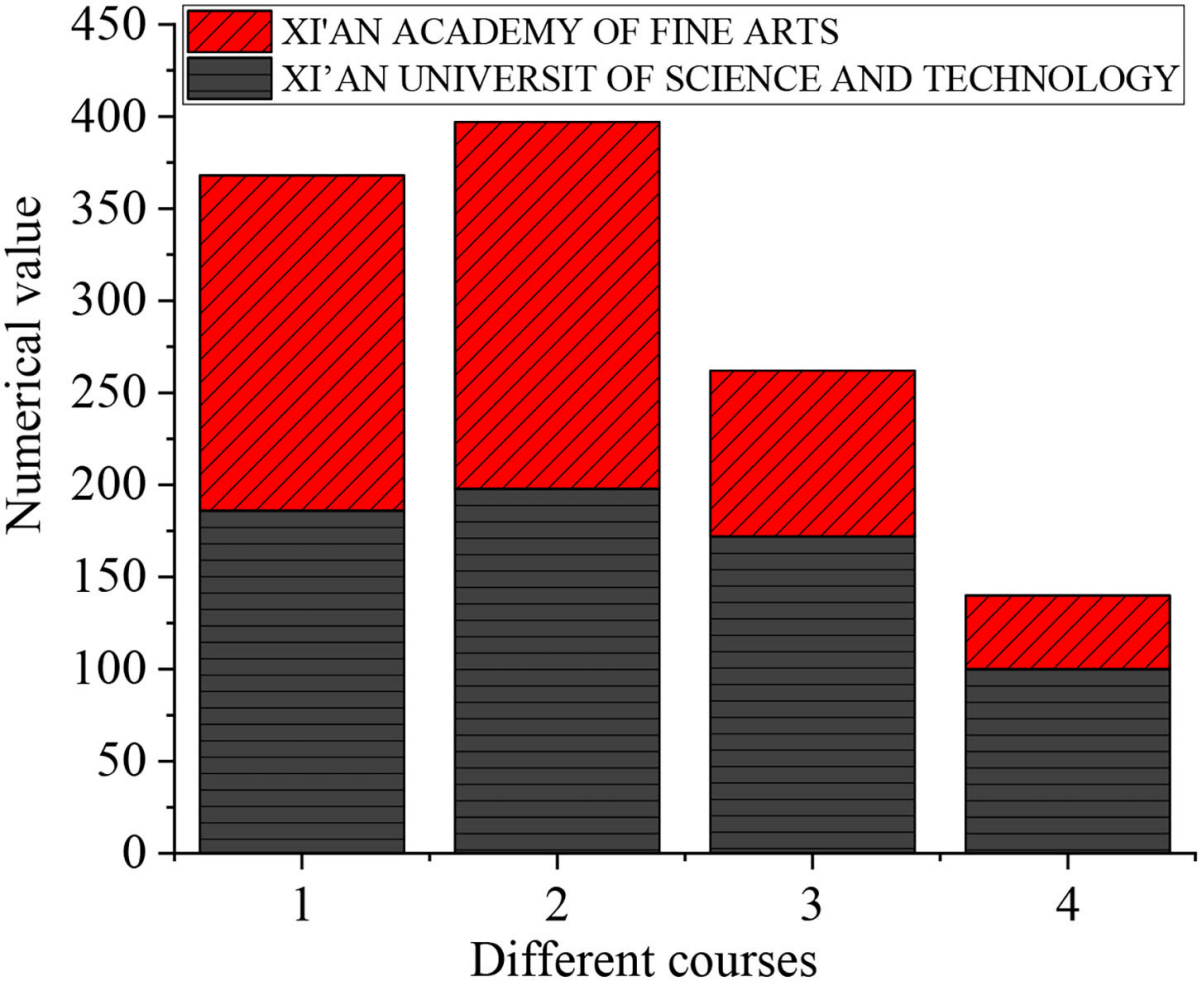
The statistical results of two colleges (1: pedagogy, 2: educational psychology, 3: art design education, 4: arts psychology).

The questionnaire data of the two colleges are statistically analyzed. Figure 3 is the result.

Figure 3 shows that in Xi'an Academy of Fine Arts and Xi'an University of Science and Technology, there are 186 and 182 students choosing pedagogy, respectively, 183 and 199 students choosing educational psychology, 172 and 90 students choosing art design education, and 100 and 40 students choosing art psychology. It shows that the students of comprehensive university have a very shallow contact with art psychology.

**Figure 4:**
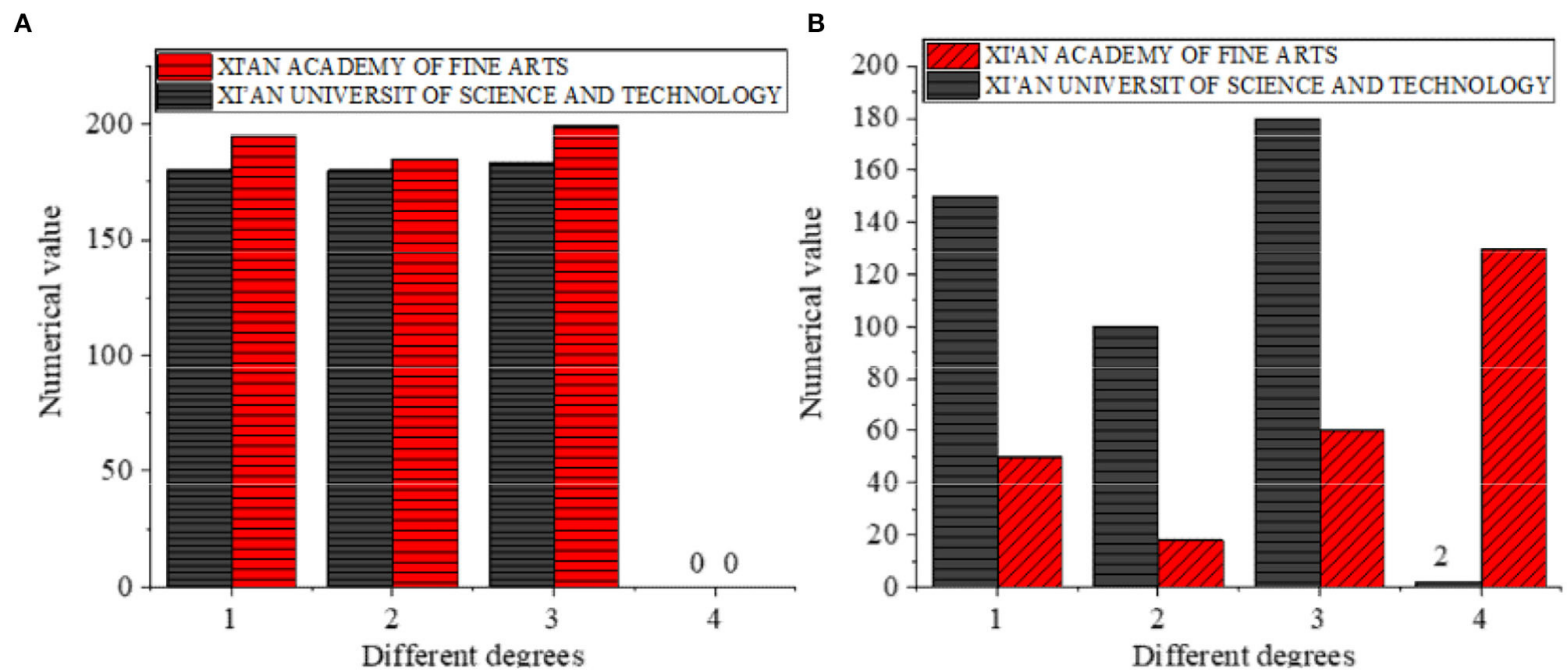
Statistical results of relevant course data contacted by students of two colleges. **(A,B)** The abscissa indicates the degree of understanding of the course, and the ordinate indicates the numerical rating of the course. (1): understand, (2): have studied, (3): have heard, (4): have never heard.

Then, the relevant curriculum data that the students of two colleges are exposed to are analyzed statistically. Figures 4A,B present the results.

According to Figure 4A, 180 and 195 students from Xi'an Academy of Fine Arts and Xi'an University of Science and Technology know about the educational psychology course, 180 and 185 students have taken the course, and all the students who participate in the survey have heard of the course. Figure 4B suggests that for art psychology, there are 150 and 50 students in Xi'an Academy of Fine Arts and Xi'an University of Science and Technology who understand the course, 100 and 18 students have taken the course, 180 and 60 students have heard of it, while 2 and 130 students have not heard of it. This shows that the basic compulsory course of professional art colleges and comprehensive universities is educational psychology, but art psychology is not a compulsory course. However, for professional art colleges, non-professional college students' understanding of art psychology is very low, and more than half of the students have not heard of the course.

### Comparison of Aesthetic Ability of Students in Different Colleges

**Figure 5:**
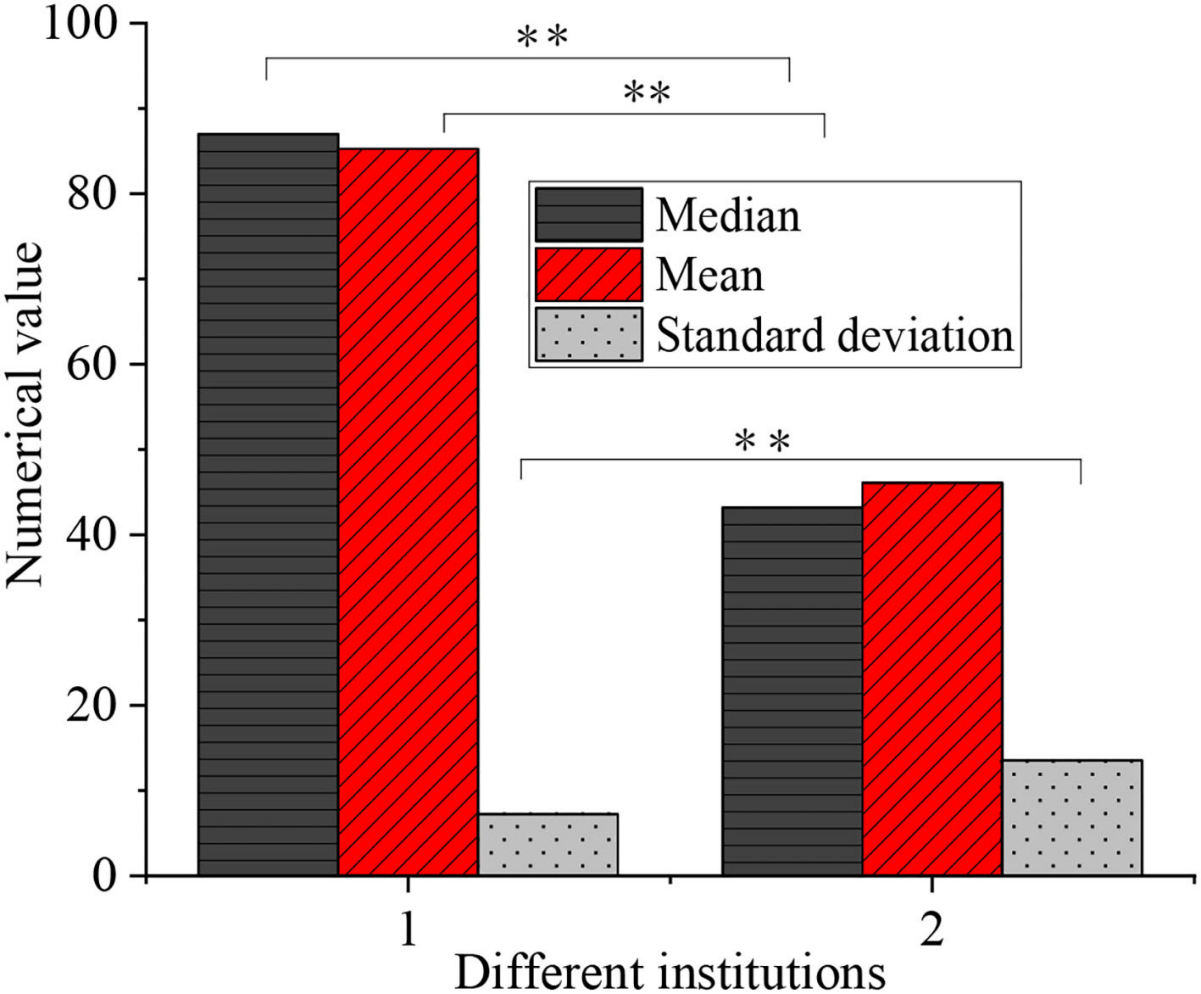
Statistical results of students' aesthetic ability in two colleges (1: Xi'an Academy of Fine Arts, 2: Xi'an University of Science and Technology, **p < 0.01).

The questionnaire data about students' aesthetic ability in two colleges were statistically analyzed, as shown in Figure 5.

Figure 5 shows that the aesthetic differences between the students of Xi'an Academy of Fine Arts and Xi'an University of Science and Technology are significant (*p* < 0.01). The students of professional fine arts colleges and comprehensive universities are groups with different aesthetic ability and aesthetic cultivation foundation. Due to the lack of art psychology and art education foundation, there is a certain gap in their aesthetic ability.

### Comparison of Life Values of Different College Students

**Figure 6:**
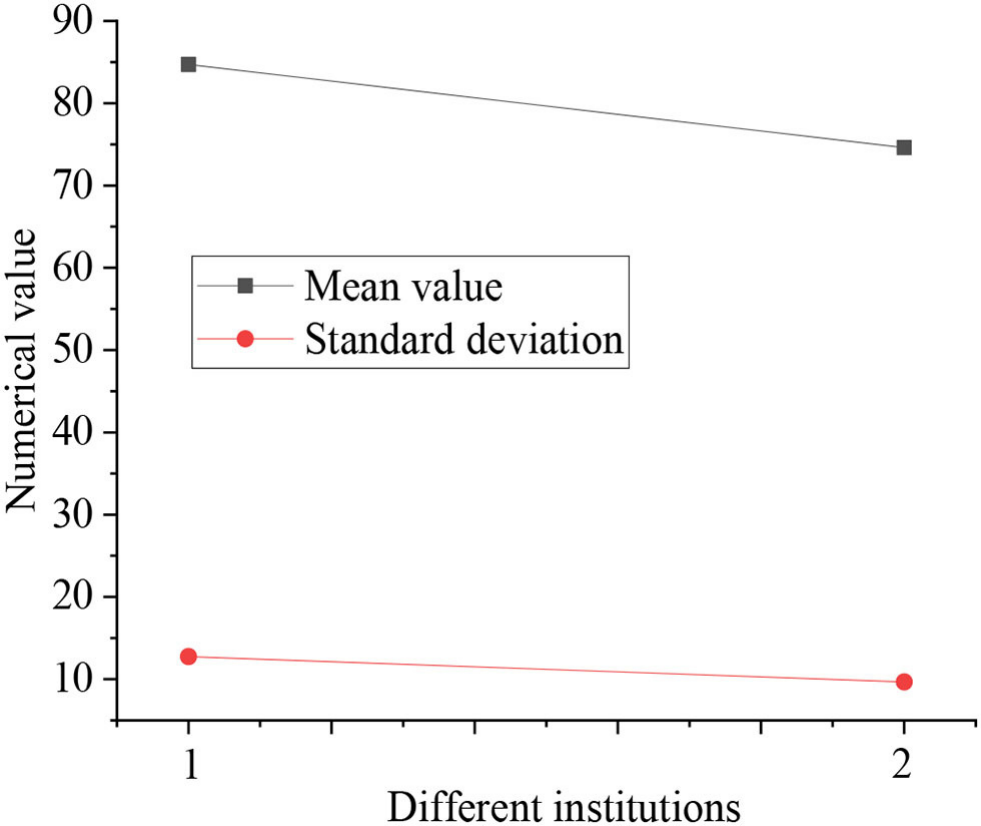
The results of the overall differences of life values between the two colleges (1: Xi'an Academy of Fine Arts, 2: Xi'an University of Science and Technology, *p* < 0.01 *t* = 3.106).

The questionnaire data about students' life values of two colleges were statistically analyzed, as shown in Figure 6.

Figure 6 shows that there is a significant difference in the values of life between the two colleges (*p* < 0.01).

**Figure 7:**
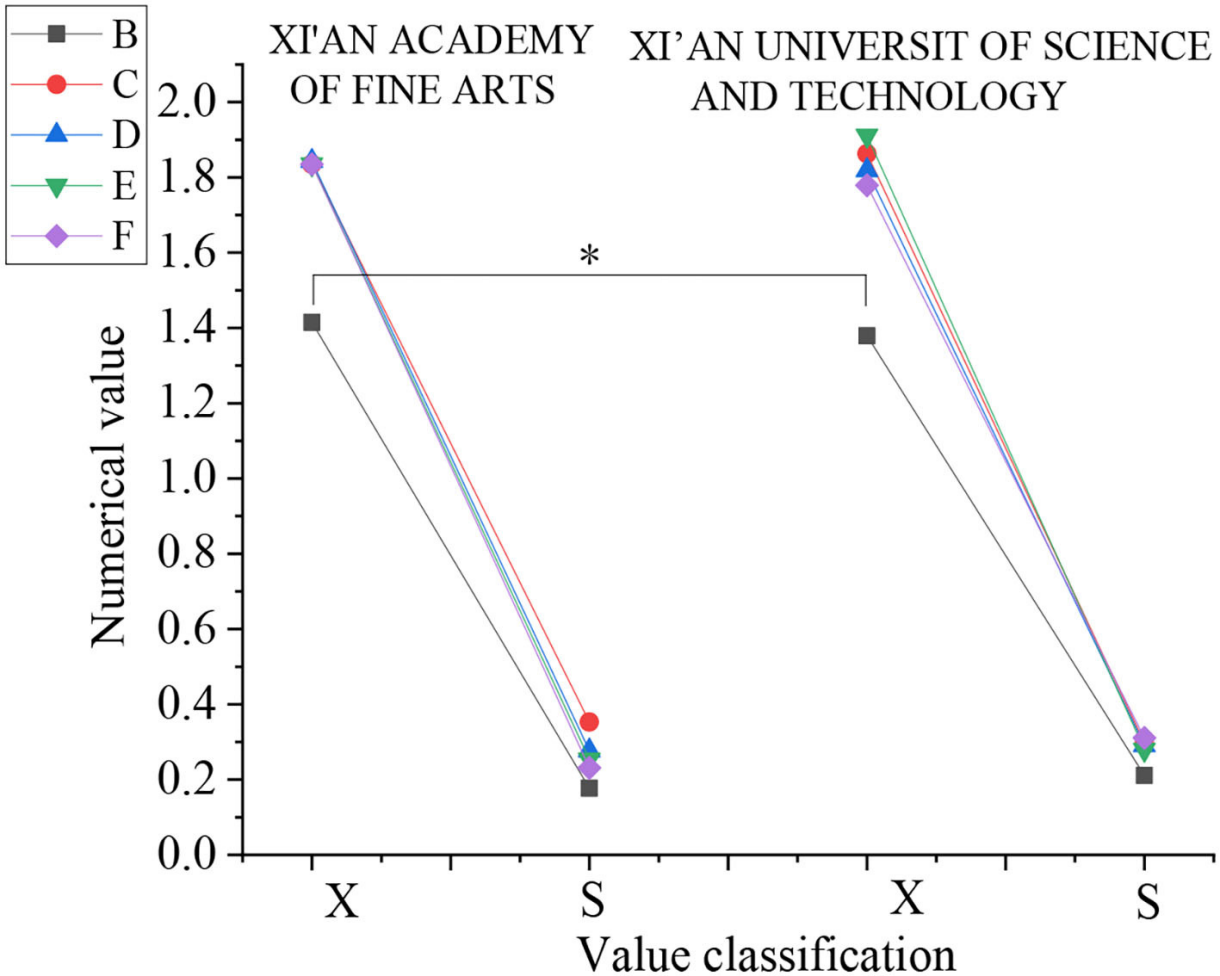
Data analysis results of “life purpose” dimension (X: mean value, S: standard deviation, B: spirit—material, C: true knowledge—fallacy, D: beauty—ugliness, E: noble—despicable, F: modern—obsolete).

The value of life has three dimensions. First, the dimension of “life purpose” is analyzed. Figure 7 is the result.

Figure 7 shows that in the five directions of “life purpose,” there is only significant difference between the two colleges in the spirit—material direction (*p* < 0.05), and there is no significant difference in the other four directions (*p* > 0.05).

**Figure 8:**
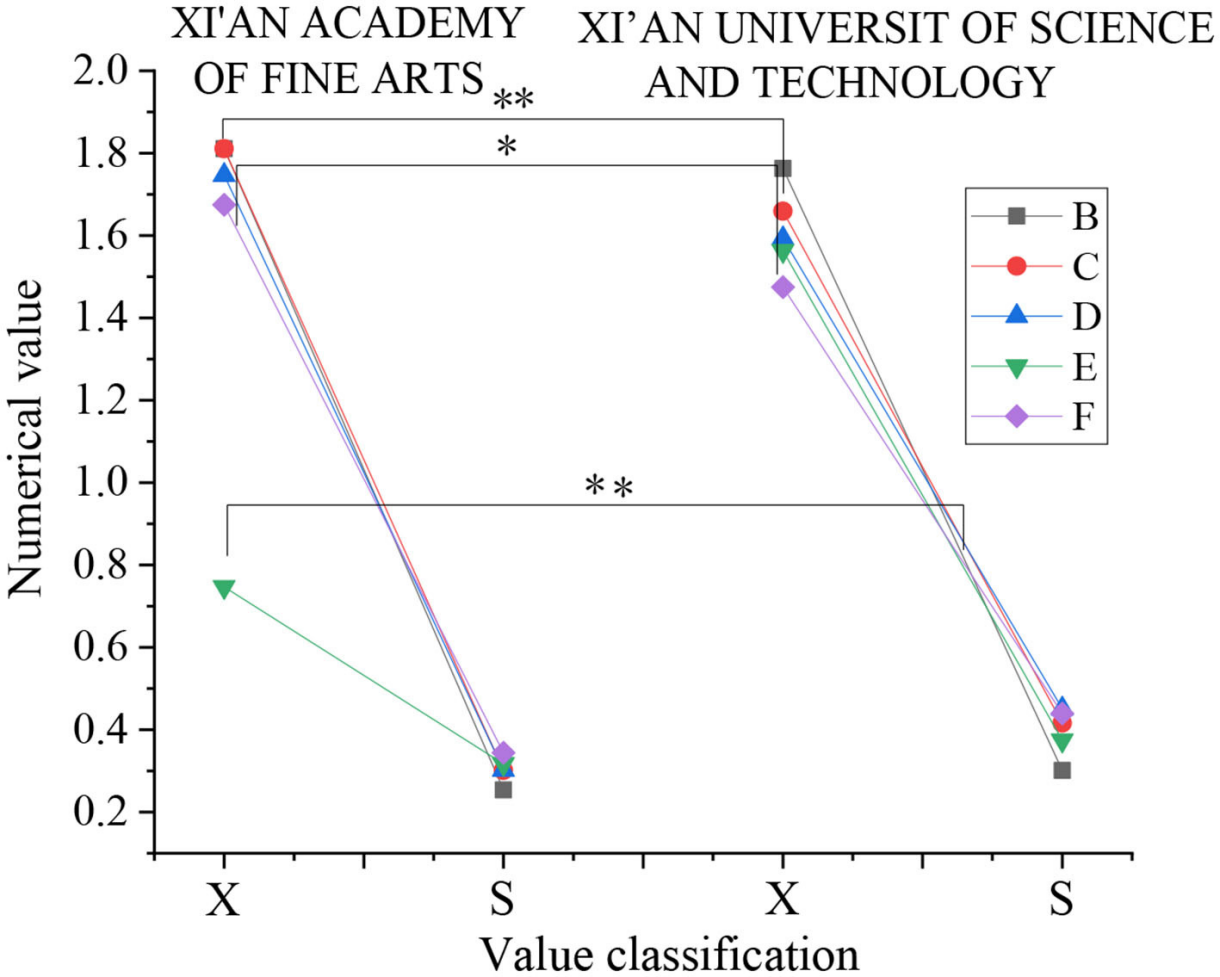
Data analysis results of “life attitude” dimension (X: mean value, S: standard deviation, B: enterprising—shrinking, C: enrichment-emptiness, D: firm—wavering, E: hope—despair, F: enthusiasm—apathy).

Then, the dimension of “life attitude” is analyzed. Figure 8 presents the results:

Figure 8 shows that in the five directions of “attitude toward life,” there are only significant differences between the two colleges in the two directions of enrichment – emptiness and enthusiasm – apathy (*p* < 0.05), while there are very significant differences between the two colleges in the direction of hope – despair (*p* < 0.01). There is no significant difference in the other two directions (*p* > 0.05).

**Figure 9:**
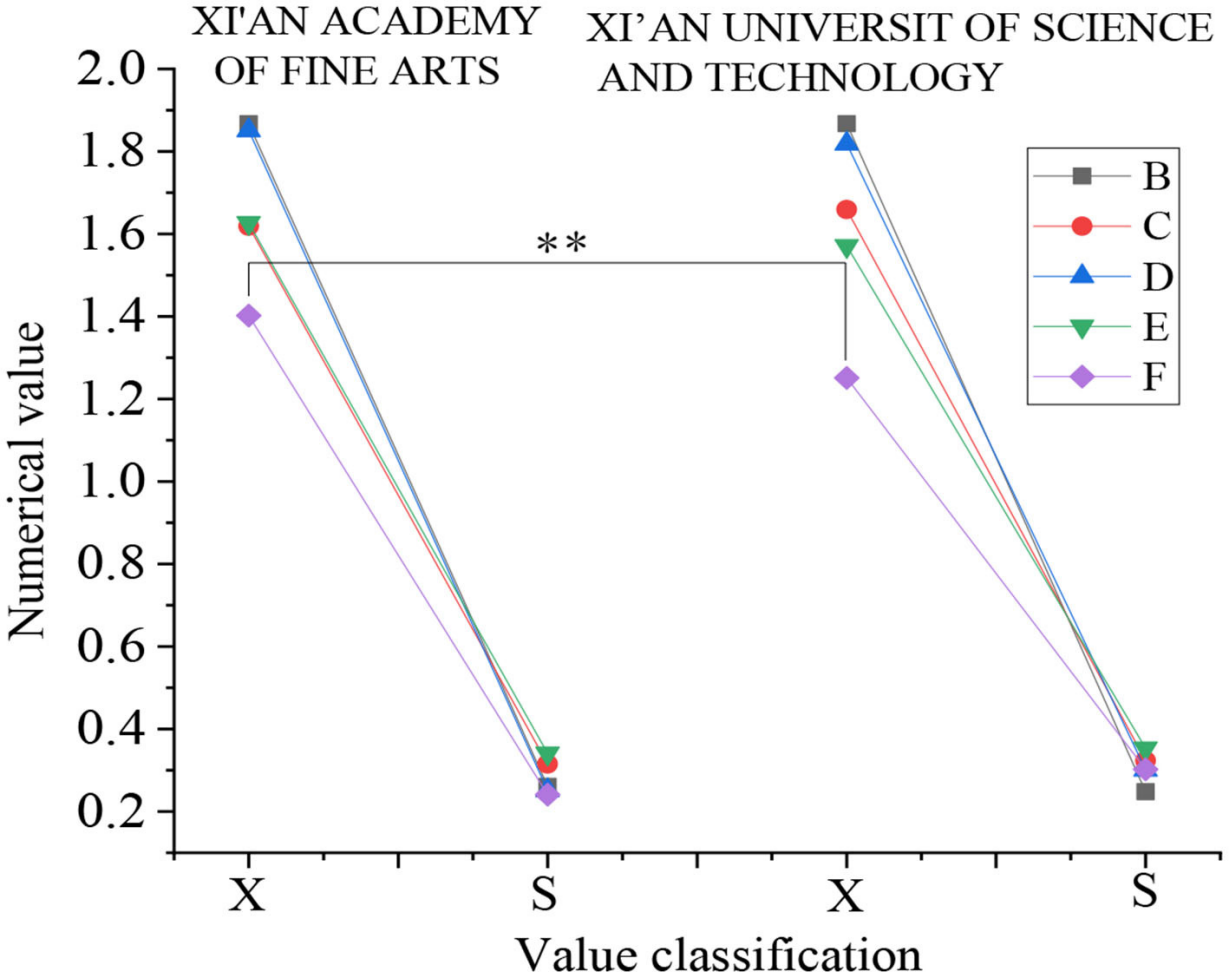
Analysis results of life style dimension data (X: mean value, S: standard deviation, B: love—hate, C: cooperation—isolation, D: effort—laziness, e: action—fantasy, F: dedication—acceptance).

Finally, “life style” dimension was analyzed, as shown in Figure 9.

Figure 9 shows that for the five directions of “life,” only the dedication – acceptance direction has a significant difference between the two colleges (*p* < 0.01), and the other four directions have no significant difference (*p* > 0.05). This shows that even if the whole system of values of life is stable for individuals, there are directions which are relatively easy to change, and these changeable directions will also promote the system to change in a positive direction.

The moral cultivation and comprehensive quality of art college students are good on the whole. They have active and sharp thinking, creativity and innovative spirit, are full of passion for life, and their pursuit of “truth, goodness and beauty” is stronger than that of non-art majors. They have prominent personality, draw a clear demarcation between whom or what to hate or love, and have strong response to social problems. They are a special group of the most active demons among college students. They are full of hope for the great rejuvenation of the Chinese nation. Based on this understanding, it is considered that the teaching and management of students in art colleges should be viewed dialectically from a rational point of view to explore a set of more appropriate teaching and management modes. It is to change the hard preaching as a more targeted education mode, strengthen the cultivation education in behavior, psychology, and learning, create a relaxed and harmonious environment for students' artistic creation and education, explore ideas, actively respond, and strive to innovate educational contents and methods.

## Conclusion

For the innovation and entrepreneurship education in the academy of fine arts, emphasis must be put on the integration with professional education, so that students can obtain perceptual experience through professional practice; the core content should focus on integrity, cultivate students' rational action, and use their professional advantages to seek the development space of the industry. This exploration creatively analyzes the theoretical basis of educational psychology and art psychology from the perspective of art education, and expounds the relationship between them. Moreover, combined with the characteristics of college students' psychological development, the objectives and significance of art psychology-related courses are analyzed. In the actual research, through the form of questionnaire, the art education of professional art colleges (Xi'an Academy of Fine Arts) and comprehensive universities (Xi'an University of Science and Technology) were investigated and analyzed in an innovative manner. The results show that the students in comprehensive universities have little contact with art psychology. Educational psychology is a basic compulsory course in professional art colleges and comprehensive universities, but art psychology is not a compulsory course. Compared with professional art colleges, non-professional college students' understanding of art psychology is very low, and more than half of the students have never heard of the course. There are significant differences in aesthetic ability and life values between comprehensive universities and professional art colleges. This suggests that educators in different colleges should carry out art design education according to students' different psychological conditions.

Based on educational psychology, the education level of art psychology is increased, so that students can form correct aesthetic standards and life values. Academy of fine arts should formulate relevant policies, management systems, and implementation measures for students' innovation and entrepreneurship, which shall be supervised and implemented by relevant departments. In addition, a perfect teaching management system should be established, relevant policies for innovation and entrepreneurship practice should be formulated, and an innovation and entrepreneurship education curriculum system should be constructed, so as to comprehensively stimulate the college students' innovation consciousness and entrepreneurial enthusiasm.

However, there are also some shortcomings. The number of research objects is limited only from two colleges, which will have a certain impact on the applicability of the research results. Therefore, in the future research plan, the scope of samples and the scope and nature of research colleges will be expanded as far as possible, and more influencing factors of modern art design education will be analyzed.

## Data Availability Statement

The raw data supporting the conclusions of this article will be made available by the authors, without undue reservation.

## Ethics Statement

The studies involving human participants were reviewed and approved by Neijiang Normal University Ethics Committee. The patients/participants provided their written informed consent to participate in this study. Written informed consent was obtained from the individual(s) for the publication of any potentially identifiable images or data included in this article.

## Author Contributions

All authors listed have made a substantial, direct, and intellectual contribution to the work and approved it for publication.

## Conflict of Interest

The authors declare that the research was conducted in the absence of any commercial or financial relationships that could be construed as a potential conflict of interest.

## Publisher's Note

All claims expressed in this article are solely those of the authors and do not necessarily represent those of their affiliated organizations, or those of the publisher, the editors and the reviewers. Any product that may be evaluated in this article, or claim that may be made by its manufacturer, is not guaranteed or endorsed by the publisher.
